# Effects of Essential Oils on Biological Characteristics and Potential Molecular Targets in *Spodoptera frugiperda*

**DOI:** 10.3390/plants13131801

**Published:** 2024-06-29

**Authors:** Júlia A. C. Oliveira, Letícia A. Fernandes, Karolina G. Figueiredo, Eduardo J. A. Corrêa, Leonardo H. F. Lima, Dejane S. Alves, Suzan K. V. Bertolucci, Geraldo A. Carvalho

**Affiliations:** 1 Department of Agriculture, Federal University of Lavras, Lavras 37203-202, Brazil; julia.assuncaooliveira@hotmail.com (J.A.C.O.); leticiafrbs@gmail.com (L.A.F.); suzan@ufla.br (S.K.V.B.); 2 Departament of Entomology, Federal University of Lavras, Lavras 37203-202, Brazil; kgfigueiredo17@gmail.com; 3 Minas Gerais Agricultural Research Company (EPAMIG), Pitangui 352650-000, Brazil; eduardo@epamig.br; 4 Exact and Biological Science Department, Federal University of São João del Rei, Sete Lagoas Campus, Sete Lagoas 35701-970, Brazil; leofrancalima@ufsj.edu.br; 5 Agronomy Course Coordination, Federal Technological University of Paraná, Santa Helena 85892-000, Brazil; dejane_bio@yahoo.com.br

**Keywords:** botanical insecticides, virtual screening, *Eugenia uniflora*, octopamine receptor

## Abstract

*Spodoptera frugiperda* control methods have proved to be inefficient, which justifies the search for new control measures. In this search for botanical insecticides for controlling *S. frugiperda*, the following were evaluated: (i) the toxicity of essential oils (EOs) from *Cinnamodendron dinisii*, *Eugenia uniflora*, and *Melaleuca armillaris*; (ii) the effect of EOs on life table parameters against *S. frugiperda*; (iii) the chemical characterization of EOs; and (iv) the in silico interaction of the chemical constituents present in the three EOs with the molecular targets of *S. frugiperda*. The EO from *E. uniflora* had the lowest LD_50_ (1.19 µg of EO/caterpillar). The major compounds bicyclogermacrene (18.64%) in *C. dinisii* and terpinolene (57.75%) in *M. armillaris* are highly predicted to interact with the octopamine receptor (OctpR). The compound 1,8-cineole (21.81%) in *M. armillaris* interacts mainly with a tolerant methoprene receptor (MET) and curzerene (41.22%) in *E. uniflora*, which acts on the OctpR receptor. Minor compounds, such as nerolidol in *C. dinisii* and β-elemene in *E. uniflora*, are highly ranked for multiple targets: AChE, MET, OctpR, and 5-HT1. It was concluded that the EO from *E. uniflora* negatively affects several biological parameters of *S. frugiperda* development and is promising as an active ingredient in formulations for controlling this insect pest.

## 1. Introduction

The fall armyworm *Spodoptera frugiperda* (JE Smith, 1797) (Lepidoptera: Noctuidae) stands out among insect pest species as a cause of great economic losses. This noctuid is a polyphagous insect capable of causing substantial damage to corn, cotton, soybean, and rice crops, among others. This species is native to the Americas but has dispersed to other continents [1,2,3,4,5,6,7].

In addition to being a pest that causes a reduction in productivity and in the quality of the final product, *S. frugiperda* is difficult to manage due to its great ability to proliferate and the numerous reports on the selection of populations resistant to the tools used for its control, such as synthetic chemical insecticides and resistant plant technology, including Bt technology [8,9,10,11,12,13,14,15]. In addition, synthetic insecticides cause harmful effects on non-target organisms, such as natural enemies, pollinators, mammals (including humans), and the environment [16,17,18].

The use of essential oils (EOs) as part of integrated pest management (IPM) for the control of *S. frugiperda* is an alternative tool to the chemical method [11,19,20,21]. Given that they exhibit faster degradation in the environment, they are often selective toward beneficial organisms and have low to moderate toxicity in mammals [22,23]. The EOs of the species *Cinnamodendron dinisii* (Schwacke) Occhioni (Canellaceae), *Eugenia uniflora* L. (Myrtaceae), and *Melaleuca armillaris* (Sol. ex Gaertn.) Sm. (Myrtaceae) were previously investigated, and great larvicidal potential was observed against *Culex quinquefasciatus* (Diptera: Culicidae); *Sitophilus zeamais* Motschulsky (Coleoptera: Curculionidae); *Drosophila melanogaster* Meigen, 1830 (Diptera: Drosophilidae); *Anopheles subpictus* Grassi, 1899; *Aedes albopictus* Skuse, 1894 and *Culex tritaeniorhynchus* Giles, 1901 (Diptera: Culicidae) [24,25,26,27,28]. Given this information, we hypothesize that these three EOs may also have the potential to control *S. frugiperda*.

In ecology and insect control studies, the use of life tables is an essential tool for analyzing survival and reproductive patterns over significant periods of time. In this way, it is possible to verify the lethal and sublethal effects of essential oils (EOs) on insect populations, with the aim of understanding their long-term impacts [29]. Another important tool today is chemoinformatics protocols that allow predictions to be made about the modes of action of the compounds present in EOs [25,30]. These in silico analyses have the potential to drive the discovery of new molecules and strategies for pest control as well as the reduction of resistance, promoting significant advances in this field. EOs have different sites of action and can, therefore, reduce the resistant populations of insects [25,30]. In addition, by allowing greater survival of natural enemies, they contribute to delaying the evolution of resistance since these organisms feed on both resistant and susceptible pest genotypes.

Thus, the objective of this study was first to evaluate the lethal and sublethal effects of the EOs of *C. dinisii*, *E. uniflora* and *M. armillaris* on *S. frugiperda*. In addition, we expanded our investigation to predict the in silico interaction of chemical constituents present in the three EOs with specific molecular targets, such as the methoprene-tolerant receptor (MET), serotonin receptor (5-HT1), ecdysone receptor (ECNr), and octopamine receptor agonist (OctpR), and inhibition of the enzyme acetylcholinesterase (AChE) of *S. frugiperda*. These targets were selected based on their importance as key proteins in insect metabolism and their involvement in the mechanisms of action of natural and synthetic substances such as extracts, EOs, and synthetic insecticides.

## 2. Results

### 2.1. Chemical Characterization of EOs

The *C. dinisii* EO presented the highest chemical complexity, with 15 compounds identified, followed by the *E. uniflora* and *M. armillaris* EOs, with 13 and 10 compounds detected, respectively. The major compounds in the *C. dinisii* EO were α-pinene (19.90%), bicyclogermacrene (18.64%), β-pinene (13.71%), and sabinene (11.53%), accounting for 63.78% of the total composition of this EO. In the *M. armillaris* EO, it was possible to identify 96.71% of the total chemical composition, where approximately 80% of this composition corresponded to the major compounds terpinolene (57.75%) and 1,8-cineole (21.81%). The major compounds in the EO of *E. uniflora* were curzerene (41.22%) and germacrene B (8.04%) (Table 1).

### 2.2. Bioassays with S. frugiperda

#### 2.2.1. Acute Toxicity of EOs to *S. frugiperda* in a Topical Application Trial

**Figure 1:**
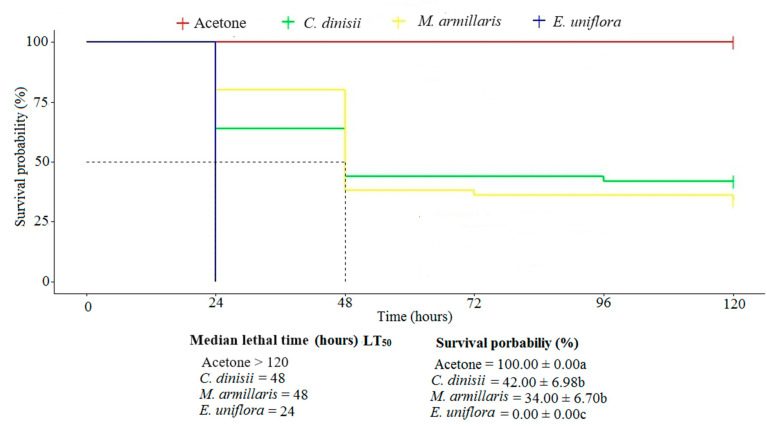
Survival analysis of *Spodoptera frugiperda* caterpillars after topical application of the essential oils from *Cinnamodendron dinisii*, *Eugenia armillaris*, and *Melaleuca armillaris* at a dose of 100 µg of EO/caterpillar. Means followed by different letters differ from each other.

The acute toxicity of EOs was evaluated by applying the EOs to the back of *S. frugiperda*. The *E. uniflora* EO was the most toxic to caterpillars of *S. frugiperda*, causing 100% mortality in the insects, and the LT_50_ (time to kill 50% of the population) was only 24 h. The EOs of *M. armillaris* and *C. dinisii* caused mortality rates of approximately 60% and 75%. Both EOs showed an LT_50_ of 48 h in *S. frugiperda* caterpillars (χ^2^ = 143; df = 3; *p* ≤ 0.05) (Figure 1).

#### 2.2.2. Chronic Toxicity of EOs to *S. frugiperda* in an Ingestion Trial

**Figure 2:**
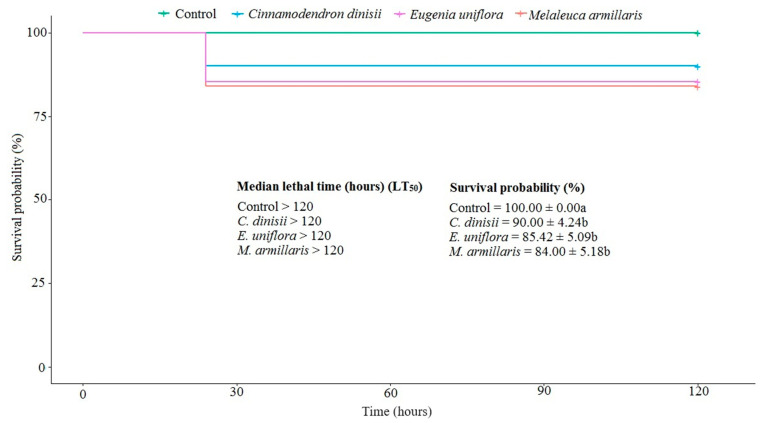
Survival analysis of *Spodoptera frugiperda* caterpillars after being fed a diet containing the essential oils of *Cinnamodendron dinisii*, *Eugenia armillaris*, and *Melaleuca armillaris* at a concentration of 1 mg of EO/mL. Means followed by different letters differ from each other.

The chronic toxicity of EOs was evaluated by an ingestion trial of the EOs to *S. frugiperda*. The EOs of *C. dinisii*, *M. armillaris*, and *E. uniflora* were slightly toxic to caterpillars of *S. frugiperda* in the ingestion assay. The mortality rates in the treatments with EOs varied between 10 and 16% during the seven days of evaluation. The confidence intervals of the negative controls overlapped, indicating a 100% probability of survival after 120 h of evaluation. The LT_50_ for all treatments was greater than 120 h (χ^2^ = 8.4; df = 3; *p* ≤ 0.04) (Figure 2). At the end of seven days after ingestion of the artificial diet containing the controls and the EOs, the weight (g) of the caterpillars was determined, and they did not differ from each other. The data is not shown instead.

#### 2.2.3. Determination of Dose-Response and Time-Response Curves

**Figure 3:**
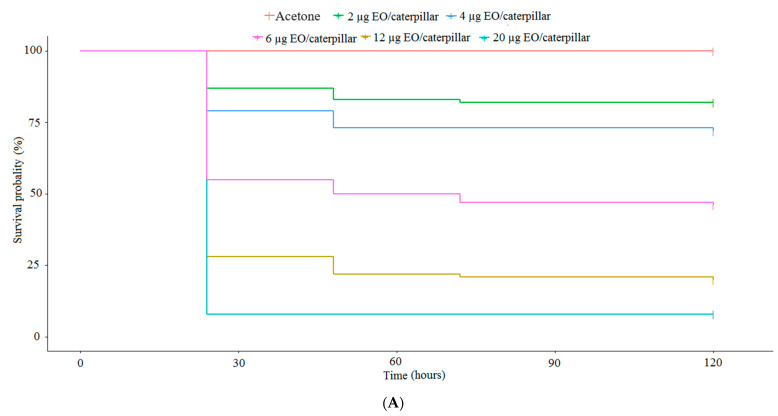

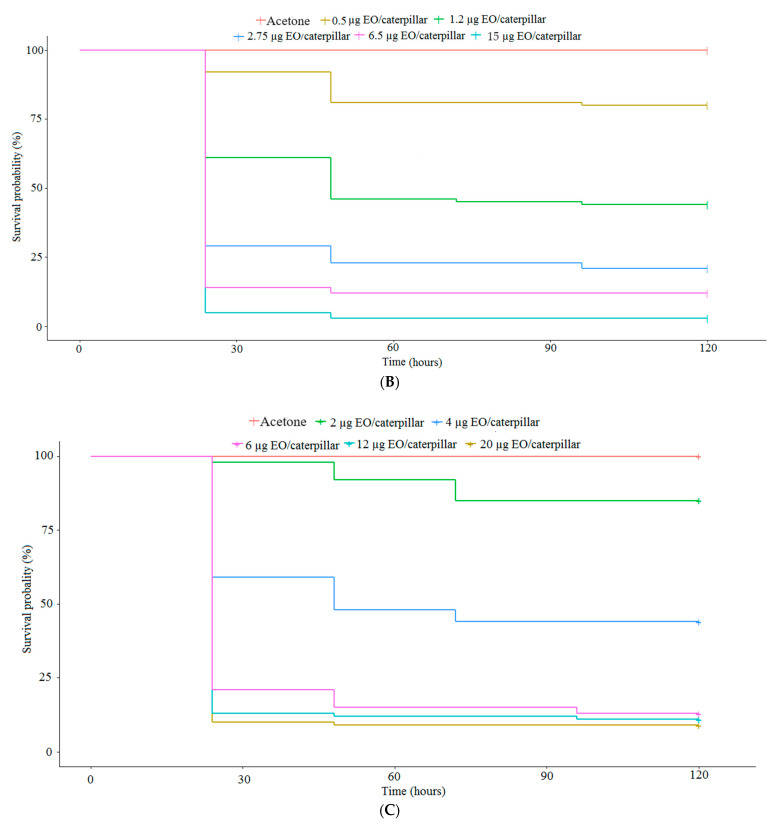
Survival of *Spodoptera frugiperda* caterpillars after topical application of essential oil doses. (**A**) *Cinnamodendron dinisii*; (**B**) *Eugenia uniflora;* (**C**) *Melaleuca armillaris*.

The mortality response was dose-dependent (Figure 3). For doses of 2.0 and 4.0 µg EO/caterpillar of *C. dinisii*, an LT_50_ > 120 h was observed with survival probabilities of 84 and 78%, respectively, with no difference between these treatments. For doses of 6.0, 12.0 and 20.0 µg EO/caterpillar, the LT_50_ was 24 h, with a survival probability of 55, 26 and 10%, respectively, where all doses differed from each other and from the other treatments (χ^2^ = 146; df = 5; *p* ≤ 0.05) (Figure 3A). The EO of *E. uniflora* was applied to the caterpillar at doses of 0.5, 1.20, 2.75 e, and 6.5 µg EO/caterpillar. The insect survival probability varied between 28 and 15% at 2.75 and 6.5 µg EO/caterpillar. At a dose of 15.0 µg EO/caterpillar, the TL_50_ was 24 h, with a survival probability of 6% (χ^2^ = 114; df = 5; *p* ≤ 0.05) (Figure 3B). Regarding the EO of *M. armillaris*, the probability of survival varied between 98, 60 and 22% at doses of 2.0, 4.0 and 6.0 µg EO/caterpillar and LT_50_ >120 h. For the highest dose tested (20.0 µg EO/caterpillar), an LT_50_ of 24 h was observed, with a survival probability of 8% (χ^2^ = 90.4; df = 5; *p* ≤ 0.05) (Figure 3C).

After estimating the LD_50_, it was found that the *E. uniflora* EO was 4.7 times more toxic than the *C. dinisii* EO and 3 times more toxic than the *M. armillaris* EO (Table 2).

#### 2.2.4. Life History and Demographic Parameters of *S. frugiperda* Caterpillar Treated with the Essential Oils of *C. dinissi*, *E. uniflora* and *M. armillaris*

**Figure 4:**
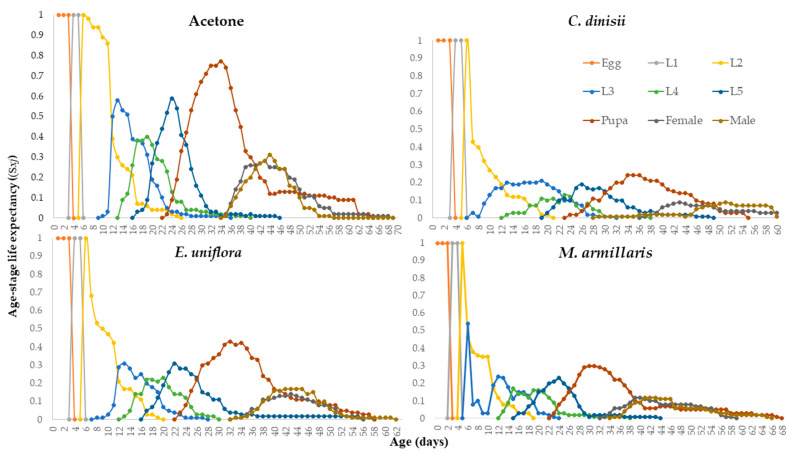
Survival rate by age-specific stage (S*_xj_*) of *Spodoptera frugiperda* in the control treatments (acetone), *Cinnamodendron dinisii*, *Eugenia uniflora*, and *Melaleuca armillaris*.

After topical application of the LD_50_ of the three EOs, it was observed that the doses of the EOs were the most lethal to the caterpillar stage (L_2_–L_5_), and the initial population was reduced by 20% by acetone, 54% by *E. uniflora* EO, 70% by *C. dinisii* EO and 66% by *M. armillaris* EO. The highest total mortality (egg-adult) was obtained with the LD_50_ of *C. dinisii* EO (78%), followed by the LD_50_ of *M. armillaris* EO (73%) and *E. uniflora* EO (65%) (Figure 4 and Table 3).

Notably, the highest mortality occurred in the L_2_ and L_3_ stages when the treatments were applied. Topical application of the LD_50_ of the three EOs affected the duration of the egg-pupal and egg-adult periods. The LD_50_ of *C. dinisii* EO increased the egg-pupal period by 0.6 days and the egg-adult period by 3.54 days compared to the control. The LD_50_ of *M. armillaris* EO also increased the egg-adult period by 2.21 days. The duration of the biological cycle of male insects was also affected by the treatments with the LD_50_ of the EOs, where the EOs of *C. dinisii* and *M. armillaris* increased the cycle duration of males by 5.69 and 4.15 days compared to the control, respectively (Table 3).

**Figure 5:**
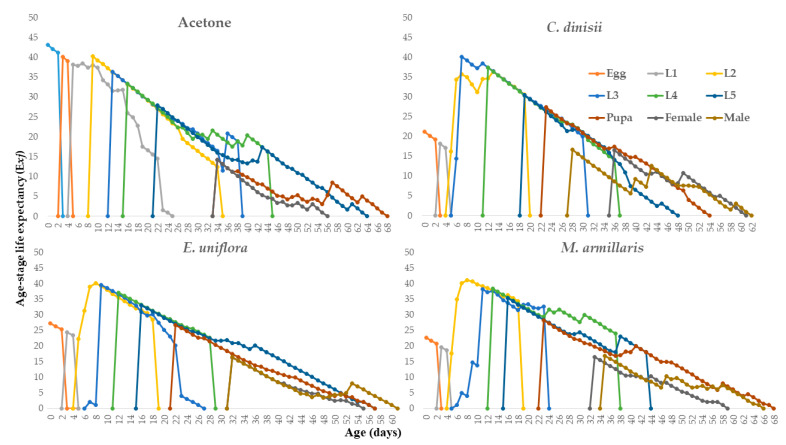
Life expectancy by age-stage (Exj) of *Spodoptera frugiperda* in the control treatments (acetone), *Cinnamodendron dinisii*, *Eugenia uniflora*, and *Melaleuca armillaris*.

The lowest age-stage survival rate (S_xj_) was observed at the caterpillar stage. The total life cycle of the insects was reduced in the treatments with the LD_50_ of the EOs of *C. dinisii*, *E. uniflora*, and *M. armillaris* at 61, 60, and 67 days. However, the EO of *E. uniflora* was the one that most negatively influenced the duration of the females’ life cycle (55 days). The females and males from the *E. uniflora* EO treatment emerged before the insects from the *C. dinisii* and *M. armillaris* EO treatments. The adults treated with the *E. uniflora* EO emerged before the adults treated with the LD_50_ of the other two EOs. This can be explained by the shorter duration of the egg-caterpillar period caused by the LD_50_ of the EO of *E. uniflora* (Figure 5).

The LD_50_ of the EO of *C. dinisii* reduced the following demographic parameters: intrinsic growth rate (r) and finite growth rate (λ). The *E. uniflora* EO reduced the average generation time (T) by 3.19 days compared to the control. The EO of *C. dinisii* increased this parameter by 9.41 days compared to the control (Table 4).

### 2.3. Virtual Screening and Computational Analysis

**Figure 6:**
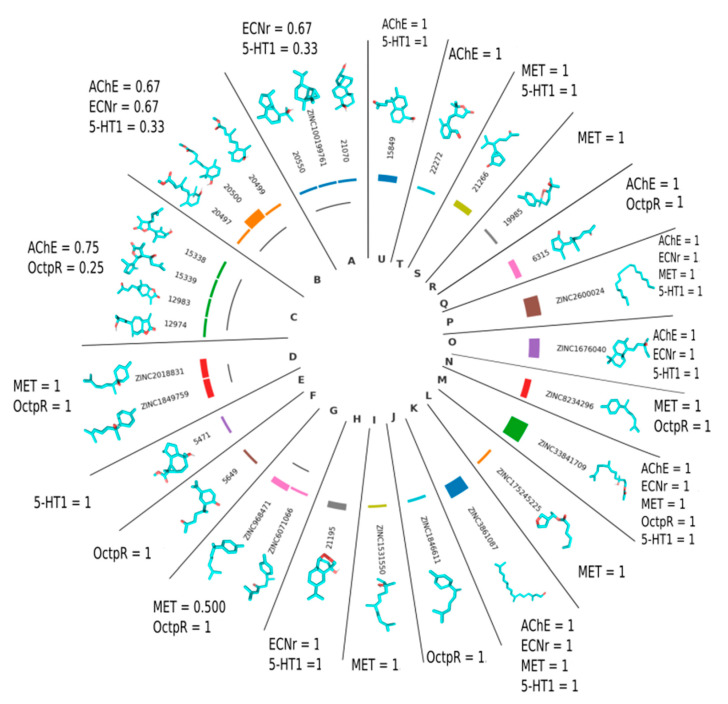
Distribution of high-scoring ligands for protein targets (methoprene-tolerant receptor (MET), serotonin receptor (5-HT1), ecdysone receptor (ECNr), octopamine agonist receptor (OctpR), and acetylcholinesterase enzyme (AChE)) of *Spodoptera frugiperda* in the clusters established by hierarchical clustering. The height of the bar represents the number of targets with a high score for the ligand. The chemical structure is shown, and the numbers represent the probability of the protein target being affected by the hierarchical cluster.

The clusters formed by the chemical similarity of the highly ranked compounds for the molecular targets of *S. frugiperda* are shown in Figure 6.

**Figure 7:**
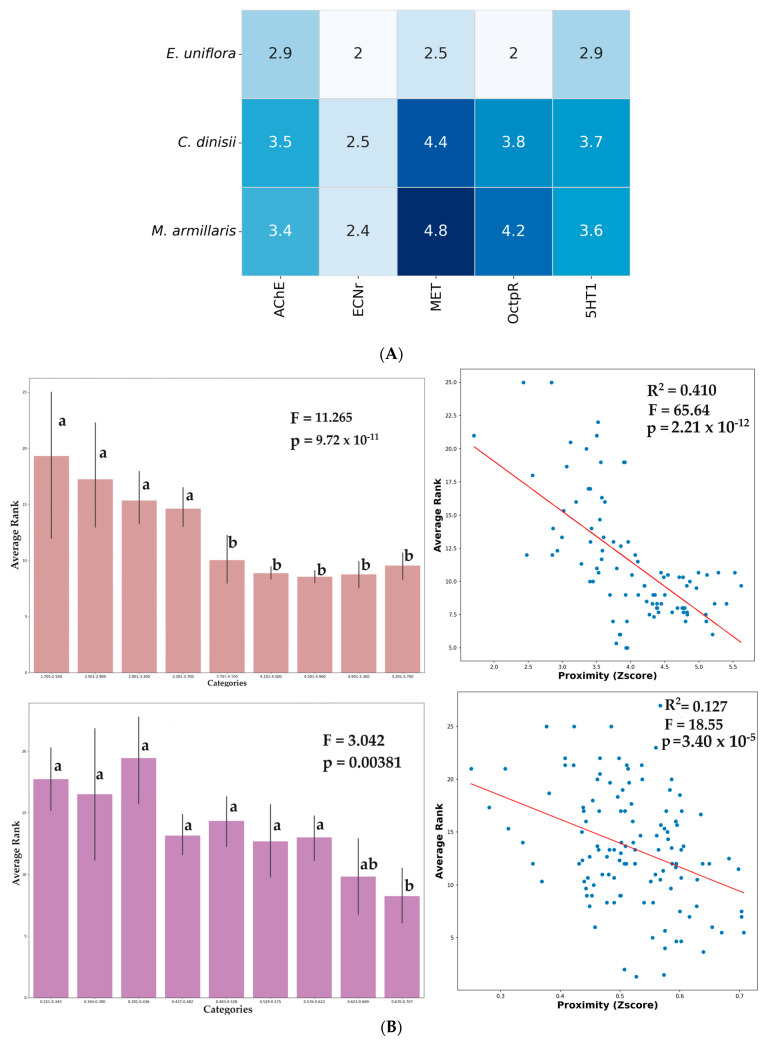
Predictive matrix of the action of the chemical compounds identified in the essential oils and regression analysis of average rank: (**A**) Predictive matrix of the action of the chemical compounds identified in the essential oils of *Cinnamodendron dinisii*, *Melaleuca armilaris*, and *Eugenia uniflora* on the targets (methoprene-tolerant receptor (MET), agonist serotonin receptor (5HT1), ecdysone receptor (ECNr), octopamine agonist receptor (OctpR), and acetylcholinesterase enzyme (AChE) of *Spodoptera frugiperda*) (**B**) Regression analysis of average rank obtained by molecular docking considering different score functions and the Euclidean metric based on chemoinformatics descriptor vectors.

Multiplying the cluster matrix by the probability matrix and, after that, by the matrix with the fraction of each EO compound obtained by CG, it could be a matrix with a prediction of the action of the three EOs on the molecular targets of *S. frugiperda*. This prediction matrix is shown in Figure 7.

Based on this prediction matrix based on the chemical similarity of the hit compound with EO compounds present in the three species’ EO, it could be observed that these three EO species interacted mainly with MET and OctpR, secondarily with AChE and 5HT1, and poorly with ECNr. Considering the three species, the *M. armillaris* EO has the highest predicted interaction, followed by *C. dinisii*, and the weaker predicted interaction of *E. uniflora* with the *S. frugiperda* targets.

**Figure 8:**
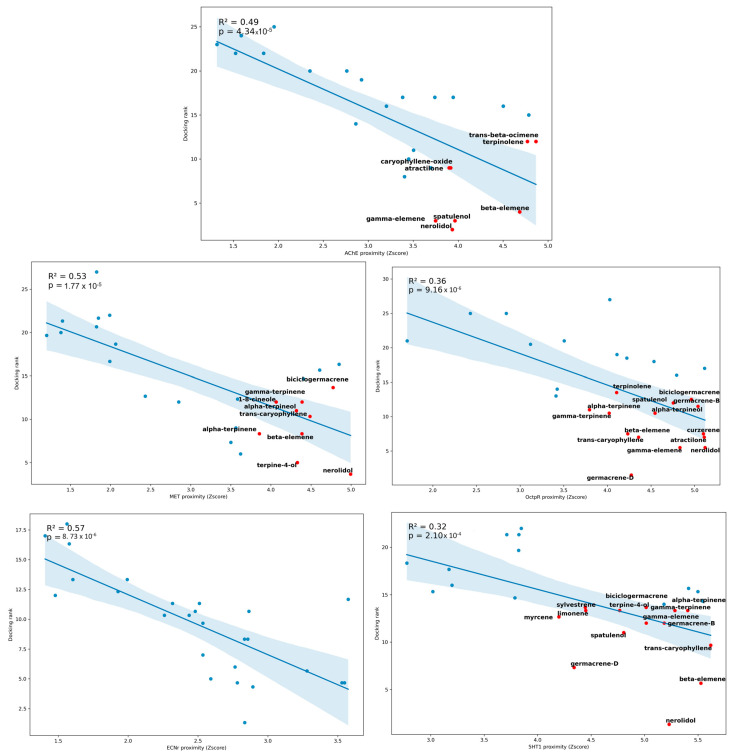
The average docking rank determined by different molecular docking score functions of the compounds from the essential oils of *Cinnamodendron dinisii*, *Melaleuca armillaris*, and *Eugenia uniflora* with the targets methoprene-tolerant receptor (MET), serotonin receptor (5HT1), ecdysone receptor (ECNr), octopamine agonist receptor (OctpR), and acetylcholinesterase enzyme (AChE) according to the scores of the selected functions.

The mean ranks determined by different docking score functions for all compounds present in the three EOs with the *S. frugiperda* targets were analyzed and are shown in Figure 8.

This figure highlights that the compounds in red are EOs compounds that comprise the features of the bar in Matrix 1 Figure 7B. In other words, EOs compounds are highly ranked by docking and present high Euclidean proximity to the chemical signature of high-scoring ligands, as presented in Figure 6. It could be inferred that nerolidol and β-elemeno are highly ranked for all molecular targets (MET, OctpR, 5HT1, and ECNr) of *S. frugiperda*. The compounds germacrene D (8.04%), curzerene (41.22%), terpinolene (57.75%), and bicyclogermacrene (18.64%) have a high prediction to interact with the OctopR. 1,8-cineole is highly concentrated in *M. amillaris* (21.81%), and bicyclogermacrene is present in *C. dinisii* (18.64%), with a signature of compounds that act on MET. Terpinolene, identified with 57.75% of the EO of *M. amillaris* by CG, had the best-predicted action on the AChE enzyme. The EOs’ compounds are, in general, poorly ranked to the ECNr target. Compounds like (e)-nerolidol and β-elemene are very indiscriminate, interacting with multiple targets.

**Figure 9:**
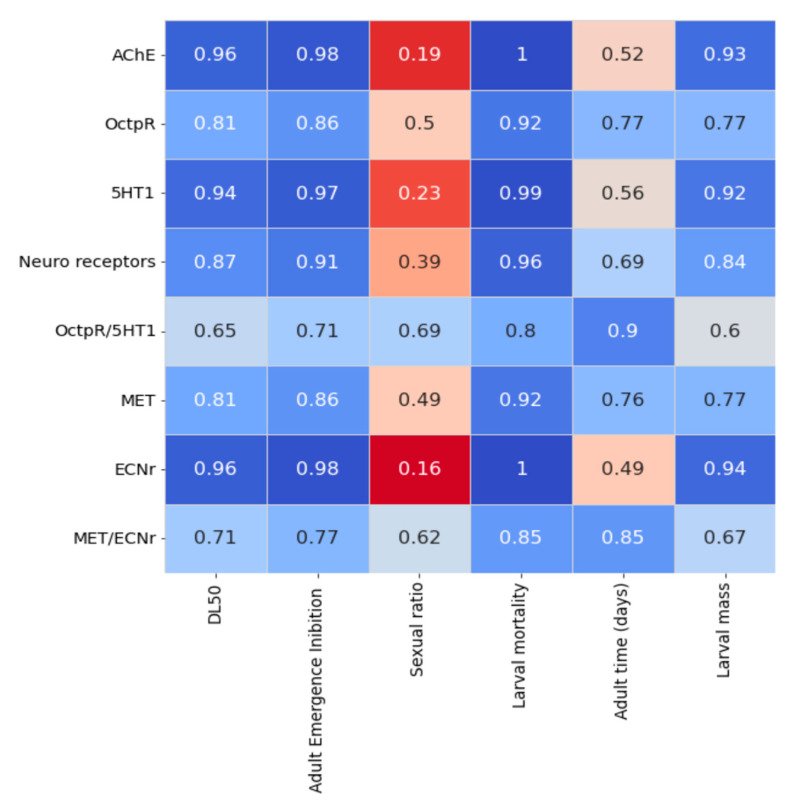
Correlogram of the signatures of the essential oils for the different molecular targets of *Spodoptera frugiperda* with the different parameters evaluated in the in vivo bioassay with the LD_50_ of the EOs of *Cinnamodendron dinisii*, *Eugenia uniflora*, and *Melaleuca armillaris*. Legend: The greater the intensity of the blue color, the more positive the correlation between the variables, with 1 being the maximum Pearson correlation value. The greater the intensity of the red color, the less positive the correlation between the variables, with 0 being the minimum Pearson correlation value.

We can see in Figure 9 the correlations between the calculated chemical signatures of the EOs for *S. frugiperda* molecular targets. Positive correlations are observed in LD_50_, adult emergence inhibition, larval mortality, and the predicted signatures of EOs to the molecular targets studied, and a poor correlation between calculated chemical signatures and sexual ratio and adult lifetime (in days).

## 3. Discussion

The EOs of *C. dinisii*, *E. uniflora*, and *M. armillaris* showed lethal and sublethal effects against *S. frugiperda*. The LD_50_ of these EOs was estimated, and the EO of *E. uniflora* showed the lowest dose, 1.19 µg of EO/caterpillar, followed by the EO of *M. armillaris* (3.66 µg/caterpillar), and *C. dinisii* (5.62 µg of EO/caterpillar). Among the sublethal effects, it was observed that all LD_50_ values caused changes in demographic parameters and in the duration of each instar. It is, therefore, inferred that the compounds present in the EOs in this study are responsible for the effects and alterations observed.

This is the first report of the toxicity of the EOs of *C. dinisii*, *E. uniflora*, and *M. armillaris* to *S. frugiperda*. However, the insecticidal activity of these EOs has been reported for other insect pests: *S. zeamais*, *D. melanogaster*, *A. subpictus*, *A. albopictus* and *C. tritaeniorhynchus* [24,25,26,27,28]. In addition, these three EOs are toxic and alter the biochemical parameters of *C. quinquefasciatus* larvae [24,25]. The potential use of Myrtaceae EOs for the control of *S. frugiperda* and other lepidopterans has been described in the literature. The EOs of the Myrtaceae *Eucalyptus staigeriana*, *Corymbia citriodora* (Hook.) KD Hill & LAS Johnson, *Syzygium aromatum* (L.) Merr. & LM Perry, *Melaleuca alternifolia* (Donzela and Betche) Cheel, *Melaleuca leucadendra* (L.), *Corymbia citriodora* (=*Eucalyptus citriodora*) and *Eucalyptus globulus* Labil. have been previously tested against *Plutella xylostella* Linnaeus 1758 (Lepidoptera: Plutellidae) [31,32,33]. In addition, *E. uniflora* EO has a repellent effect on the caterpillar and reduces the weight and oviposition of *Diaphania hyalinata* Linnaeus, 1767 (Lepidoptera: Crambidae) [34]. In contrast, for the EOs of the family Canellaceae, no studies have investigated their potential for controlling *S. frugiperda*.

The EOs evaluated were more efficient than others considered promising for the control of *S. frugiperda* because they had an LD_50_ that ranged from 1.19 to 5.62 µg of EO/caterpillar. These values were higher than those of the EOs of the species *Lippia sidoides* Cham. (Verbenaceae), with an LD_50_ of 3.21 mg EO/g caterpillar, the majority of which was thymol (73.3%); *Hyptis marrubioides*, with an LD_50_ of 18.49 µg of EO/g caterpillar and *Ocimum basilicum*, with an LD_50_ of 38 and 21 µg of EO/caterpillar, both of the family Lamiaceae, whose major constituents were β-thujone (41.5%) and linalool (35.68%), respectively; *Lippia microphylla* Cham. (Verbenaceae) with an LD_50_ of 104.52 mg/mL and *Vanilloamopsis arborea* Backer (Asteraceae) with an LD_50_ of 172.86 mg/mL, the majority of which were 1,8-cineole (73.29%) and α-bisabolol (94.17%), respectively, demonstrating the potential of the EOs tested herein for the control of *S. frugiperda* [35,36,37].

In the assessment of the lethal and sublethal effects of the LD_50_ of the three EOs were observed high mortality rates for these doses compared to the control. It was also observed that the EO doses caused sublethal effects, such as changes in the duration of developmental stages (caterpillar, pupal, and adult), sex ratio, longevity (days) of females and males, and population parameters (intrinsic growth rate (r) and finite growth rate (λ)) of *S. frugiperda*. Although lethal effects are more visible and immediate, sublethal effects can have equally significant consequences, often more subtle but potentially long-lasting. Changes in developmental stages, sex ratio, and longevity of insects can directly affect population structure and population dynamics. Changes in the sex ratio can affect the reproductive success of the population, while changes in longevity can influence the rate of population growth [29].

Furthermore, understanding the sublethal effects is essential for developing more effective and sustainable pest control strategies. By considering not only immediate mortality but also the long-term effects on behavior, reproduction, and intra-specific interactions, it is possible to design more precise and targeted control measures, minimizing negative impacts on the environment. Finally, sublethal effects have important implications for the management of insecticide resistance, as understanding these effects is essential to avoid the selection of resistant pest populations in the long term. In short, research into the sublethal effects of EOs in pest control is fundamental for a holistic and sustainable approach to insect management, promoting not only the effectiveness of control but also the conservation of ecosystems and the prevention of resistance.

The observed results corroborate those described in the literature, where in one study, the authors observed that the compounds limonene and (E)-anethole, both present in EOs, caused adverse effects on the reproductive parameters of *S. frugiperda*, especially testicular apoptosis [38]. In addition, the morphological changes caused by the EO doses in *S. frugiperda* caterpillars and pupae caused the death of these individuals. This likely occurred due to malformation or blockage of the spiracles that prevented the insects from breathing, which is one of the possible mechanisms of action of EO compounds [39,40].

For the EO of *C. dinisii*, the major compounds were identified as bicyclogermacrene, α-pinene, β-pinene, and sabinene. For the EO of *M. armillaris*, the major compounds were terpineol and 1,8-cineole. The EO of *E. uniflora* had curzerene as the major compound. The literature mentions that the compounds α- and β-pinene, myrcene, germacrene B and D, 1,8-cineole, curzerene, trans-β-elemenone, γ-elemene, among others, are responsible for the insecticidal activity of EOs [24,25,26,27,28,38,39,40]. In addition, the minority constituents of EOs may also be responsible for the insecticidal activity [25]. Thus, the synergism between the EO molecules may explain the diversity of EO action mechanisms [41].

Due to the chemical complexity of EOs, they can cause lethal and sublethal effects in insects through different mechanisms of action. The mechanisms of action of EOs include AChE inhibition, activation of octopamine receptors, and changes in protein, hydrogen peroxide (H_2_O_2_), and reduced glutathione (GSH) levels [24,25,26,27], [42]. In this context, in silico investigation allowed the prediction that all the compounds identified in the three EOs interact with the molecular targets of *S. frugiperda*. However, this prediction allowed us to observe that some compounds have different interactions with molecular targets and, therefore, may act on multiple or specific targets.

The computational prediction indicated that nerolidol and β-elemene are a multitarget compound highly interacting with all *S. frugiperda* targets studied. The multitarget effect found for nerolidol agrees with that described in the literature; in insects, it also shows reported effects like repellency, caterpillar mortality, prolongation of the caterpillar and pupal stages, inhibition and malformation of pupae, and ovicidal activity [43,44,45]. The major EO compound of *M. armillaris*, terpinolene (57.75%), exhibited a high predicted interaction with AChE enzyme and insect Octopamine receptor. The insect Octopamine receptor (OctpR) is a G-protein that acts in the neurohormonal inset response [46]. As demonstrated by Thompson et al. (1990) [47], the agonist action on this receptor leads to the suppression of biosynthesis of juvenile hormone (JH) in the insect *Corpus Allatum*. JH is a very important insect hormone involved in insect metamorphosis [48], and alterations in its concentration are responsible for causing developmental abnormalities [49]. In muscles, OctpR regulation plays an important role in muscle fibers by altering basal tone and modulating mating behavior [50,51,52,53,54]. Additional in vivo studies are needed to confirm the effects of EO compounds on the sensory, behavioral, and hormonal physiology of *S. frugiperda*.

The nuclear receptor methoprene-tolerant (MET) when triggered by JH or analog molecules invokes a transcriptional signal that leads to the retention of juvenile form and regulates the insect metamorphosis [48,49,55]. Thus, differences in the duration of the caterpillar stage of *S. frugiperda* can be explained by the presence of a compound that interacts with the OctpR and MET receptors.

Terpinolene has a high concentration in *M. armillaris* (57.75%) and a high chemical signature and is highly ranked to interact with the AChE enzyme. The AChE enzyme is responsible for the hydrolysis of acetylcholine in cholinergic synapses, where its inhibition leads to insect death due to nervous system exhaustion [56]. As observed in this study, the complex mixtures of bioactive molecules, EOs of *C. dinisii*, *E. uniflora*, and *M. armillaris*, have different mechanisms of action on *S. frugiperda*. Furthermore, the synergism between the molecules may be an important aid against resistant-selection insect populations [41].

It has been observed that the action of the EOs on AChE and 5-HTP1 is apparently due to the combined contribution of various compounds present at lower concentrations. These results corroborate those reported by Oliveira et al. (2023) [25]. Although it is generally expected that the compounds in higher quantities are responsible for the biological activity of EOs, the analysis reveals that, in the case of the EOs studied, none of the compounds at high concentrations significantly affect AChE and 5-HTP1. Therefore, it is inferred that the synergism between molecules at low concentrations is what enables the high activity of the EOs concerning these targets.

The results of the present study showed that the EOs of *C. dinisii*, *E. uniflora*, and *M. armillaris* are toxic to *S. frugiperda*. The in silico results support the explanation for an EO compound’s multitarget mechanism of action in the insect. However, efforts are needed to elucidate these mechanisms of action by conducting biochemical studies with these EO compounds that show a highly predicted interaction with molecular targets of *S. frugiperda*.

In addition, the present study highlighted the importance of estimating the sublethal effects of the LD_50_ of EOs because the applications of the products in the field are often not homogeneous due to several factors, such as plant architecture, terrain slope, and the availability of specialized equipment. Notably, among the EOs evaluated in this study, only the *E. uniflora* EO was sold on the market. This species is a fruit of great economic importance in the food, cosmetic, and pharmaceutical industries [57,58]. The specie *E. uniflora* orchards are typically managed with maintenance pruning, with the leaves considered a waste byproduct, which can be used by companies that distill this EO. Thus, *E. uniflora* EO is an excellent candidate botanical insecticide to control *S. frugiperda*. In addition to having the highest toxicity among the other tested EOs, this EO offers economically viable advantages and a well-structured production chain.

## 4. Materials and Methods

### 4.1. EOs Production and Chemical Characterization

Fresh leaves of the species *C. dinisii*, *E. uniflora* and *M. armillaris* were collected at the campus of the Federal University of Lavras (UFLA) (21°14′43″ S; 44°59′59″ W), Lavras, Minas Gerais, Brazil, in the morning between August and November 2021. The plant material was herborized, identified, and incorporated into the collection of the PAMG Herbarium (Empresa de Pesquisa Agropecuária de Minas Gerais-EPAMIG, Belo Horizonte, Minas Gerais, Brazil) under records numbered 58650 for *C. dinisii*, 58800 for *M. armillaris* and 58799 for *E. uniflora*.

The fresh leaves of *C. dinisii*, *E. uniflora* and *M. armillaris* were chopped into fragments of approximately 1 cm^2^ and then subjected to steam distillation in a Marconi MA480 distiller until there was no more condensation [25]. The chromatographic parameters for the chemical analyses of the EOs of *M. armillaris* and *E. uniflora* were the same as those described in the literature [24]. For the EO of *C. dinisii*, the procedure is described in the literature [25]. The analyses were performed in triplicate, and the results were expressed as the mean normalized peak area greater than 1% ± standard deviation (n = 3).

### 4.2. Rearing of S. frugiperda

The *S. frugiperda* caterpillars were maintained on an artificial diet [59]. Adults of *S. frugiperda* were fed 10 g/L aqueous honey solution. For all bioassays, only caterpillars from the second oviposition by adults kept in the laboratory were used. The insects were reared and maintained in an acclimatized room under the following conditions: temperature of 25 ± 2 °C, relative humidity of 70 ± 10%, and photophase of 12 h.

### 4.3. Bioassays with S. frugiperda

#### 4.3.1. Acute Toxicity of EOs to *S. frugiperda* in a Topical Application Trial

The EOs (100 µL) from the leaves of *C. dinisii*, *E. uniflora* and *M. armillaris* were solubilized in acetone (100 µL) and applied topically (1 µL) to the back of 72-h caterpillar using an automatic UNISCIENCE^®^ micropipette (0.1–2.5 µL). Each caterpillar received a dose of 100 µg of EO and was placed individually in a glass tube plugged with hydrophobic cotton containing a pre-standardized piece of artificial diet (2 g). The bioassay was conducted in a completely randomized design with 50 replicates per treatment; the experimental plot consisted of a single caterpillar that was individually maintained. The negative control consisted of acetone. Insect survival was assessed every 24 h for 120 h, and this bioassay was repeated twice on different days, thus constituting a biological replicate.

#### 4.3.2. Chronic Toxicity of EOs to *S. frugiperda* in an Ingestion Trial

The EOs (10 mg) from the leaves of *C. dinisii*, *E. uniflora* and *M. armillaris* were solubilized in 1% Tween 80 aqueous solution (100 mL) plus rhodamine dye (250 µg) and added to the artificial diet (100 mL). The dye was used to visually ensure the complete incorporation of the EOs in the artificial diet. This dye was not toxic to *S. frugiperda* caterpillars in the preliminary assays. Thus, the EOs were incorporated into the diet at a concentration of 1 mg EO/mL. Diet pieces with an average weight of 2 g were transferred to glass tubes, where one 48-h caterpillar was inoculated. The bioassay was conducted in a completely randomized design with 50 replicates per treatment, and the experimental plot consisted of a single caterpillar maintained individually. Insect survival was assessed every 24 h for 168 h. The weight of the surviving caterpillars was measured on the seventh day of evaluation, and this bioassay was repeated twice on different days.

#### 4.3.3. Determination of Dose-Response and Time-Response Curves

The EOs from the leaves of *C. dinisii*, *E. uniflora* and *M. armillaris* showed acute toxicity to *S. frugiperda* only in the topical application test (Section 4.3.1). Thus, doses capable of causing mortality varying between 20 and 80% of the insects were selected using the results of previous tests and calculations of arithmetic progression [60]. For *C. dinisii* and *M. armillaris*, doses of 2.0, 4.0, 6.0, 12.0, and 20.0 µg EO/caterpillar and for *E. uniflora*, doses of 0.50, 1.20, 2.75, 6.50 and 15.0 µg EO/caterpillar were used. The bioassays and the evaluation of caterpillar mortality were conducted as described in Section 4.3.1.

#### 4.3.4. Life History Table and Demographic Parameters of *S. frugiperda* Caterpillar Treated with the EOs of *C. dinisii*, *E. uniflora* and *M. armillaris*

Approximately 500 eggs from the second oviposition by adults kept in the laboratory were separated and monitored daily until the caterpillars hatched. Then, the caterpillars were individually placed in tubes containing a piece of artificial diet until they reached 72 h of age. Each EO was solubilized in acetone at a dose equivalent to the LD_50_ (*C. dinisii*–5.62 µg EO/caterpillar; *M. armillaris*–3.66 µg EO/caterpillar and *E. uniflora*–1.19 µg EO/caterpillar). Next, 400 caterpillars (100 caterpillars per treatment) were topically treated with 1 µL aliquots of the LD_50_ of each EO and acetone. After topical application, the caterpillars were placed in glass tubes containing a 2 g piece of artificial diet. The tubes were plugged with hydrophobic cotton. The experimental design was completely randomized, with 100 replicates per treatment; the experimental plot consisted of a single caterpillar that was maintained individually. The negative control consisted of acetone. Insect survival was evaluated every 24 h until the last day of life of the last experimental individuals. The diets were replaced for the caterpillar as needed.

For the study of biological aspects, the following variables were evaluated: duration of the egg, caterpillar, pupal, and adult stages, and number of males and females in the population, as described in the literature [61,62]. Based on these data, a life and fertility table was prepared using the TWOSEX-MSChart software (Version 2020.01.12), and the following parameters were evaluated: the biological parameters used were age-stage-specific survival rate (Sxj), age-specific survival rate (lx), age-stage life expectancy (exj), intrinsic rate of increase (r), finite rate of increase (λ) and mean generation time (T) [63]. The life table considers the averages of the parameters of survival, life expectancy, and fertility until the moment when it reaches age x and stage j.

### 4.4. Statistical Analyses

The normality of the data was assessed using the Shapiro–Wilk and Bartlett normality tests. The survival data over time were subjected to survival analysis using the Kaplan–Meier estimator with the survival statistical package [64]. The survival curves were analyzed using the pairwise test. The median lethal time (LT_50_), i.e., the time needed to cause 50% mortality in the insects in each treatment, was also calculated. The data were subjected to logit regression analysis using R^®^ software [65].

The life history data for males and females were analyzed based on the age-stage, two-sex life table theory [66]. The means and standard errors of reproductive parameters were estimated using the bootstrap method and estimated for 100,000 individuals [67,68,69,70]. Differences in parameters between treatments were analyzed using the paired bootstrap test based on the confidence interval using the TWOSEX-MSChart program for Windows [62,71,72,73,74].

### 4.5. Virtual Screening and Computational Analysis

The prediction of the action of EOs on molecular targets of *S. frugiperda* [(methoprene-tolerant receptor (MET), serotonin receptor (5-HT1), ecdysone receptor (ECNr), octopamine receptor agonist (OctpR), and acetylcholinesterase enzyme (AChE))] was performed using the protocol for virtual screening, as described by Corrêa et al. [30].

Protein models of each molecular target of *S. frugiperda* were obtained using a homology modeling protocol based on crystallographic models available in the Protein Data Bank [75], and the primary amino acid sequences of the proteins of the *S. frugiperda* targets were recovered in UniProt [76]. Tertiary structure models were constructed using the SWISS-MODEL platform, as described in the literature [25,30].

Molecular docking simulations were performed, and scoring functions for each target were selected based on enrichment curves (ECs) and receiver operating characteristic curves (ROCs), as described by Corrêa et al. [30]. These curves made it possible to determine the optimal set of ranking functions to recover the biologically active ligands for each target [25,30].

After the virtual screening, the scoring results for each target were compiled, and the best ligands were identified and grouped using hierarchical clustering with 21 groups, with all groups considered chemically cohesive and well-defined using an agglomerative hierarchical clustering algorithm. Two matrices were constructed to establish a metric that relates the compounds of the essential oils determined by CG data to the chemical signature of the hits ligands clusterized, a first name probability matrix that indicates the fraction of ligands of a cluster that affects a particular target, and the specificity matrix, which evaluates the fraction of ligands that affect each target and belong to each cluster, as previously described [30]. This procedure allowed for the evaluation of the relationship between the chemical constituents of the EOs and the different molecular targets of *S. frugiperda*.

To predict the interaction between the EO constituents and the molecular targets, the probability and specificity matrices were multiplied by the values of the proximity matrix calculated based on Euclidean distances between vectors in a Cartesian space constructed using the z-score of molecules chemoinformatics descriptors, resulting in two matrices that evaluate the influence of the EO components on the target: matrices 1 and 2 of Figure 6. To choose the best prediction matrix, a Pearson correlation analysis was performed between these values and the average score rankings obtained in molecular docking using the selected scoring functions (Figure 6). Next, the predicted values were categorized into classes of 0.1 to 0.1, and an analysis of variance was performed, followed by Tukey’s post-hoc test, to compare the means of the ranked scores between the different categories and determine whether these means were the same or different. Analysis of variance was used to determine whether the observed differences between the categories were statistically significant. These results are presented in Figure 6, and this protocol has been published previously [30].

## 5. Conclusions

The EOs of *C. dinisii*, *E. uniflora* and *M. armillaris* were toxic to *S. frugiperda* since they caused sublethal effects influencing the demographic and reproductive parameters. Notably, the *E. uniflora* EO was the most toxic to *S. frugiperda*, predictively due to neurotoxic activity via enzymatic inhibition of AChE, associated with the molecular interaction in this target of the minority components such as germacrene D and spathulenol. The results of the present study indicate that the interaction with AChE is possibly due to the synergistic effects of these and other compounds present at low concentrations. The results described here open perspectives for new biochemical studies in vivo using enzymes and receptors of this pest because there are still many questions to be answered to clarify the mechanisms of action. In addition, it is possible to conduct safety, feasibility, and selectivity studies of these EOs for the natural enemies of *S. frugiperda* for use in IPM programs of *S. frugiperda*. This comprehensive approach can provide valuable insights into the development of *E. uniflora* oil as a potentially active component in effective control strategies against *S. frugiperda*.

## Figures and Tables

**Table 1:** Chemical composition of the essential oils of *Cinnamodendron dinisii*, *Eugenia armillaris* and *Melaleuca armillaris*.

No.	Compound	RRI ^a^	RRI ^b^	Area (%) ^c^ ± SD		
				*C. dinisii*	*E. uniflora*	*M. armillaris*
1	α-pinene	932	932	19.90 ± 0.16	nd	nd
2	Sabinene	969	972	11.53 ± 0.15	nd	nd
3	β-pinene	974	976	13.71 ± 0.09	nd	nd
4	myrcene	991	988	1.72 ± 0.00	nd	1.27 ± 0.01
5	α-phellandrene	1005	1002	nd	nd	2.20 ± 0.00
6	α-terpinene	1016	1014	nd	nd	1.09 ± 0.00
7	sylvestrene	1027	1025	2.00 ± 0.00	nd	nd
8	limonene	1028	1024	nd	nd	2.99 ± 0.01
9	1,8-cineole	1030	1026	2.54 ± 0.01	nd	21.81 ± 0.04
10	(Z)-β-ocimene	1036	1032	3.71 ± 0.00	nd	nd
11	(E)-β-ocimene	1045	1044	3.60 ± 0.00	1.14 ± 0.03	nd
12	γ-terpinene	1058	1054	1.27 ± 0.02	nd	2.97 ± 0.01
13	terpine-4-ol	1076	1077	1.31 ± 0.02	nd	2.98 ± 0.01
14	terpinolene	1090	1086	nd	nd	57.75 ± 0.17
15	ni	1101	-	nd	nd	1.03 ± 0.00
16	α-terpineol	1190	1173	nd	nd	2.62 ± 0.02
17	β-elemene	1390	1389	nd	6.00 ± 0.03	nd
18	(E)-caryophyllene	1416	1417	5.07 ± 0.11	2.53 ± 0.02	nd
19	γ-elemene	1432	1434	nd	2.07 ± 0.01	nd
20	germacrene D	1478	1480	nd	2.59 ± 0.04	nd
21	bicyclogermacrene	1495	1500	18.64 ± 0.57	nd	nd
22	curzerene	1497	1499	nd	41.22 ± 0.04	nd
23	germacrene B	1554	1559	nd	8.04 ± 0.07	nd
24	(E)-nerolidol	1564	1565	1.06 ± 0.04	nd	nd
25	spathulenol	1575	1577	2.09 ± 0.07	2.37 ± 0.02	nd
26	caryophyllene oxide	1581	1582	1.08 ± 0.07	nd	nd
27	atractilone	1658	1657	nd	1.17 ± 0.05	nd
28	ni	1690	-	nd	4.86 ± 0.12	nd
29	germacrone	1694	1693	nd	5.17 ± 0.05	nd
30	ni	1728	-	nd	4.50 ± 0.11	nd
31	ni	1743	-	nd	3.03 ± 0.06	nd
Number of identified compounds				15	13	10
Total area (%)				88.23	84.69	96.71

RRI ^a^: Relative retention indices calculated against n-alkanes series (C_8_–C_20_) on the HP-5 MS column by elution order; RRI ^b^: Relative retention indices on an apolar column reported in the literature. Area (%) ^c^: average of the relative percentage area of the chromatographic peaks above 1%. SD, standard deviation (*n* = 3). ni: not identified. nd: not detected or percentage of area below 1%.

**Table 2:** Lethal doses of 25, 50, and 90% of the *Spodoptera frugiperda* population when subjected to treatment with the essential oils of *Cinnamodendron dinisii*, *Eugenia uniflora*, and *Melaleuca armillaris*.

Essential Oil	N	X^2^	p	b	e	DL_25_ (µg of EO/Caterpillar)	DL_50_ (µg of EO/Caterpillar)	DL_90_ (µg of EO/Caterpillar)
*C. dinisii*	100	4.22	0.234	−1.79	5.62	3.04 ± 0.26	5.62 ± 0.34	19.16 ± 2.34
*E. uniflora*	100	7.15	0.0672	−1.69	1.19	0.62 ± 0.06	1.19 ± 0.09	4.35 ± 0.54
*M. armillaris*	100	36.82	0	−1.85	3.66	2.02 ± 0.20	3.66 ± 0.24	12.03 ± 1.32

**Table 3:** Mean values (±SE) of life history traits (development time, longevity, and total life cycle) of *Spodoptera frugiperda* after treatment with essential oils from *Cinnamodendron dinisii* (5.62 µg EO/caterpillar), *Eugenia uniflora* (1.19 µg EO/caterpillar), and *Melaleuca armillaris* (3.66 µg EO/caterpillar).

Parameter	Stages	Acetone		*C. dinisii*		*E. uniflora*		*M. armillaris*	
		N	Mean ± SE	N	Mean ± SE	N	Mean ± SE	N	Mean ± SE
Development time (days)	Egg	100	3.00 ± 0.00a	100	3.00 ± 0.00a	100	3.00 ± 0.00a	100	3.00 ± 0.00a
	L_1_	100	2.00 ± 0.00a	100	2.00 ± 0.00a	100	2.00 ± 0.00a	100	2.00 ± 0.00a
	L_2_	84	7.67 ± 0.31b	37	6.86 ± 0.71b	51	7.39 ± 0.38b	100	3.51 ± 0.35a
	L_3_	84	5.11 ± 0.26a	34	8.41 ± 0.59b	48	4.33 ± 0.23c	34	4.32 ± 0.28d
	L_4_	83	3.84 ± 0.15a	34	3.74 ± 0.27ab	48	4.12 ± 0.24b	34	4.74 ± 0.43b
	L_5_	80	5.30 ± 0.11a	30	5.63 ± 0.23a	46	5.63 ± 0.15a	34	5.79 ± 0.23a
	Pupa	67	11.94 ± 0.27a	22	12.14 ± 0.47a	35	11.83 ± 0.37ab	27	11.26 ± 0.20b
	Egg-Pupa	67	38.40 ± 0.48b	22	39.00 ± 0.00a	35	37.63 ± 0.58b	27	38.22 ± 0.67b
Longevity (days)	Adult	67	10.49 ± 0.52a	22	11.27 ± 1.09a	35	10.89 ± 0.66a	27	12.89 ± 1.27a
Life cycle (days) *	Female	35	49.57 ± 1.08a	9	52.44 ± 2.46a	15	48.40 ± 1.06a	14	50.00 ± 1.80a
	Male	32	48.16 ± 0.55a	13	53.85 ± 1.92b	20	48.60 ± 0.88a	13	52.31 ± 1.99b
	Egg-Adult	67	48.90 ± 0.62a	22	52.44 ± 2.46b	35	48.51 ± 0.67ab	27	51.11 ± 1.80b

Means in the same line followed by different letters differ from each other (*p* < 0.05). Differences between treatments were obtained using the paired Bootstrap test with 100,000 replicates. N = number of specimens in each development phase. L_1_ = 1st instar caterpillar, L_2_ = 2nd instar caterpillar, L_3_ = 3rd instar caterpillar, L_4_ = 4th instar caterpillar, L_5_ = 5th instar caterpillar. * Average total life history for males and females in days, only for insects that became adults.

**Table 4:** Population parameters (±SE) of *Spodoptera frugiperda* in different treatments with the LD_50_ of the essential oils of *Cinnamodendron dinisii*, *Eugenia uniflora*, and *Melaleuca armillaris*.

Demographic Parameter	Acetone	*C. dinisii*	*E. uniflora*	*M. armillaris*
Intrinsic growth rate (r)	0.04 ± 0.01a	0.01 ± 0.01b	0.05 ± 0.02a	0.04 ± 0.02a
Finite rate of growth (λ)	1.04 ± 0.01a	1.01 ± 0.01b	1.04 ± 0.02a	1.03 ± 0.02a
Average generation time (T)	46.90 ± 0.06b	56.31 ± 0.001a	43.71 ± 0.096c	44.63 ± 4.52bc

Averages on the same line followed by different letters are significantly different at *p* < 0.05. Differences between treatments were obtained using the paired Bootstrap test with 100,000 replicates.

## Data Availability

Data are contained within the article.
